# In vitro & in vivo investigation of the silicon nitride ceramic hip implant’s safety and effectiveness evaluation

**DOI:** 10.1186/s13018-021-02884-7

**Published:** 2022-02-12

**Authors:** Xiangpeng Kong, Xiaosu Hu, Wei Chai

**Affiliations:** 1 Senior Department of Orthopedics, The Fourth Medical Center of PLA general hospital, No. 51 Fucheng Road, Haidian, Beijing, 100853 China; 2 Black Ceramic Medical Technology Co., Ltd, No. 269, Dongjin Road, Wuzhong, Suzhou, 215000 China

**Keywords:** Silicon nitride, Ceramic femoral head, Hip replacement, In vitro & in vivo experiments

## Abstract

**Background:**

With regard to the ceramic hip joint implant, given the concerns in ceramic about the alumina brittleness and zirconia instability, is there any alternative material solution for the orthopedic implant? Beyond the metastable oxide ceramics, along the echelon of advanced technical ceramics, looking at the non-oxide ceramic, the silicon nitride could be an excellent candidate for the joint implant’s application. The purpose of this study is to investigate the safety, effectiveness and to demonstrate the potential of this silicon nitride hip implant.

**Methods:**

According to the related ISO (International Organization for Standardization) standards, a series of in vitro (nine) & in vivo (five) tests, which had been accomplished for the aforementioned aim. Especially, the total hip replacement in pigs had been achieved, as per the authors’ knowledge, this is the first time to apply the THA (Total Hip Arthroplasty) in the big animal.

**Results:**

Refer to the ISO 6474-2, in comparison with the current monopolized German product, this silicon nitride ceramic hip implant has high strength, high hardness, excellent fracture toughness, lower density, better wear resistance, good biocompatibility, inherent stability, corrosion resistance and bioactivity, bone integration capability.

**Conclusions:**

This silicon nitride ceramic will be an admirable alternative solution with superior comprehensive property that can withstand the toughest conditions in the most demanding applications like in orthopedic and beyond.

## Introduction

With the increasing trend of Chinese aging population and the continuous improvement of life expectancy and living standards, people's awareness of healthy life has changed, currently Total Hip Arthroplasty (THA) is gradually admitted and accepted in China, it is also usually recognized as one of the most successful modern surgery [1].

Meanwhile, with regard to the avascular necrosis of the femoral head, the demands from the relative young patients have been raised due to the alcoholic or sportive traumata or genetic predilection. As the last resort, THA will restore these patients' health [2].

A safe, effective and durable hip implant is desirable consequently.

In terms of the material improvement, compared with John Charnley’s metal femoral head [3], the introduction of ceramic in THA was a great initiation since almost five decades ago [4].

Considering the appreciated excellent biological behavior, no metal ion release, no known pathogenic reaction to particles, excellent wettability, resistant to third-body wear, significantly low taper corrosion etc*.*, thanks to these inherent proprieties of ceramic, more than 18 million oxide ceramic components are sold since 1974 by the monopolized German giant [5].

However, the poor toughness is the biggest demerit of the alumina ceramic [6], in order to solve the problem of alumina brittleness, zirconia, one of the highest strength ceramics, has been wildly used as femoral head since 1985 [7, 8].

Nevertheless, this metastable zirconia ceramic for medical use were suspended on August 14, 2001 by Saint-Gobain Desmarquest (Vincennes, France) and a Medical Device Recall Notice was issued one month later by the FDA (U. S. Food and Drug Administration) [9] due to the catastrophic experience with Prozyr® femoral head produced by this world’s largest supplier of medical-grade stabilized zirconia ceramic [10].

The root cause of this failure is the inherent zirconia phase transformation. By a stress corrosion mechanism, the initial phase transformation of isolated grains occurs on the machined and polished surfaces, where is not only the femoral head bearing surface, but also the inner taper surface in contact with the femoral stem. The zirconia transforms from metastable tetragonal to monoclinic phase, this phase change results a volumetric expansion in the grains that induces a compressive stress increase and leads to the propagation of the micro cracks and surface roughened [11].

Under more severe environment, especially with presence of moisture, like in the human body, this zirconia intrinsic aging process will transform more aggressively to the monoclinic phase with catastrophic consequence: increased wear, grain pull-out and generation of particle debris even through the premature failure [12].

Given the concerns about the monolithic alumina brittleness and zirconia instability, to counteract these deficiencies, different approaches have been proposed like developing alumina–zirconia composites: ZTA (Zirconia Toughened Alumina) or replacing yttria with other additives such as ceria to stabilize zirconia.

Although zirconia transformation toughening effect is well appreciated, it is also exceedingly complex and still not totally understood.

Clarke et al*.* [11] and Chevalier [12] tried to pierce the veil of mystique surrounding zirconia. The potential effect of different process stages on microstructure of zirconia and consequently on phase transformation and aging were summarized. And it is remarkable that that ageing could be significant in the alumina-3Y-TZP (Yttria-stabilized Tetragonal Zirconia Polycrystal) composite above 16 vol% zirconia. This critical content was related to the percolation threshold above which a continuous path of zirconia grains allowed transformation to proceed. The latter author also emphasized that, for alumina–zirconia composite, the presence of zirconia aggregates, especially if the zirconia is stabilized with yttria, should be avoided [12].

From some orthopedic surgeons’ point of view, it is unsatisfactory to implant a material into the body, which is not fully stable.

In this respect, beyond the metastable oxide ceramics, along the echelon of advanced technical ceramics, looking at the non-oxide ceramic, the silicon nitride could be a suitable alternative material solution for joint implants.

The purpose of this paper is to investigate the silicon nitride hip implant’s safety and effectiveness via in vitro and in vivo experiments then to demonstrate the applicability and potential of this silicon nitride ceramic in the orthopedic domain.

## Materials and methods

In this study, a series of in vitro (nine) & in vivo (five) tests had been designed, 5 in vitro tests were descriptive, the rest other 4 in vitro tests and 5 in vivo tests were analytic. And, the level of evidence is Level I since the 5 in vivo tests were all randomized controlled trials.

The approval was obtained from the Institutional Review Board of Chinese PLA General Hospital prior to performing the study.

### Specimens tested

**Fig. 1:**
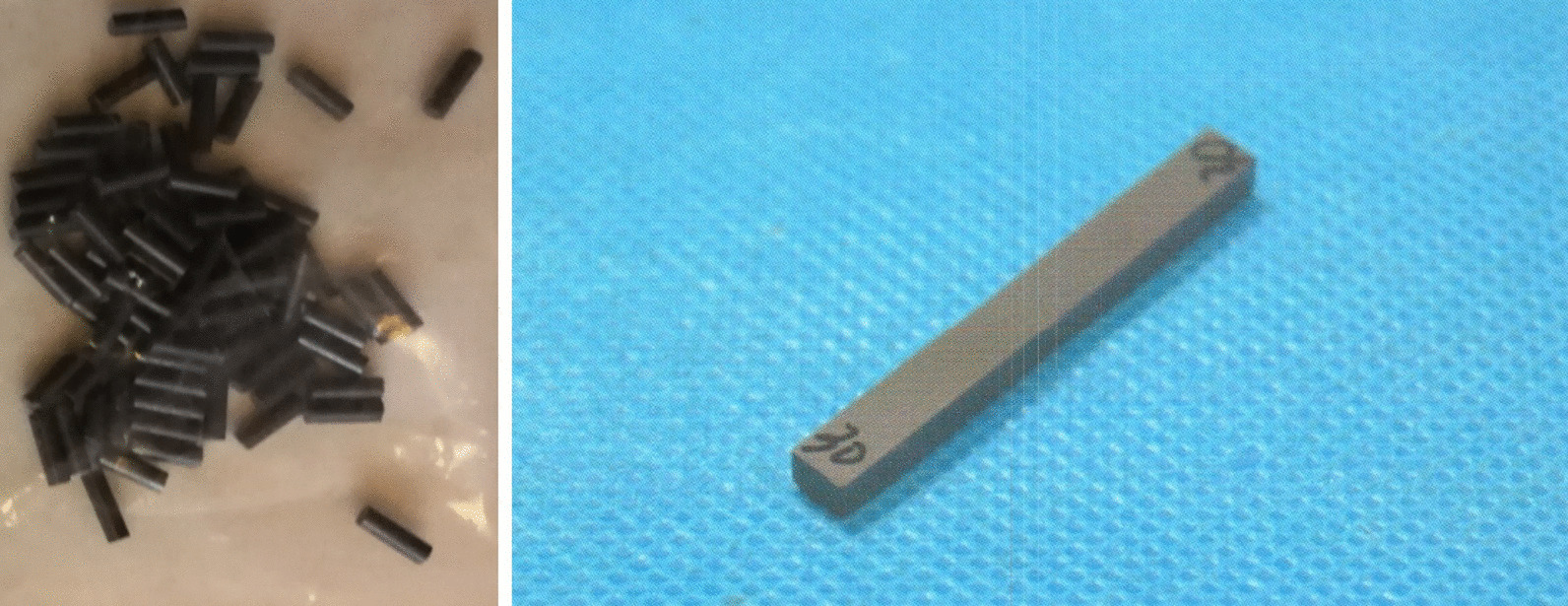
Sample bars (Φ2 * 6 mm & 3 * 4 * 50 mm)

**Fig. 2:**
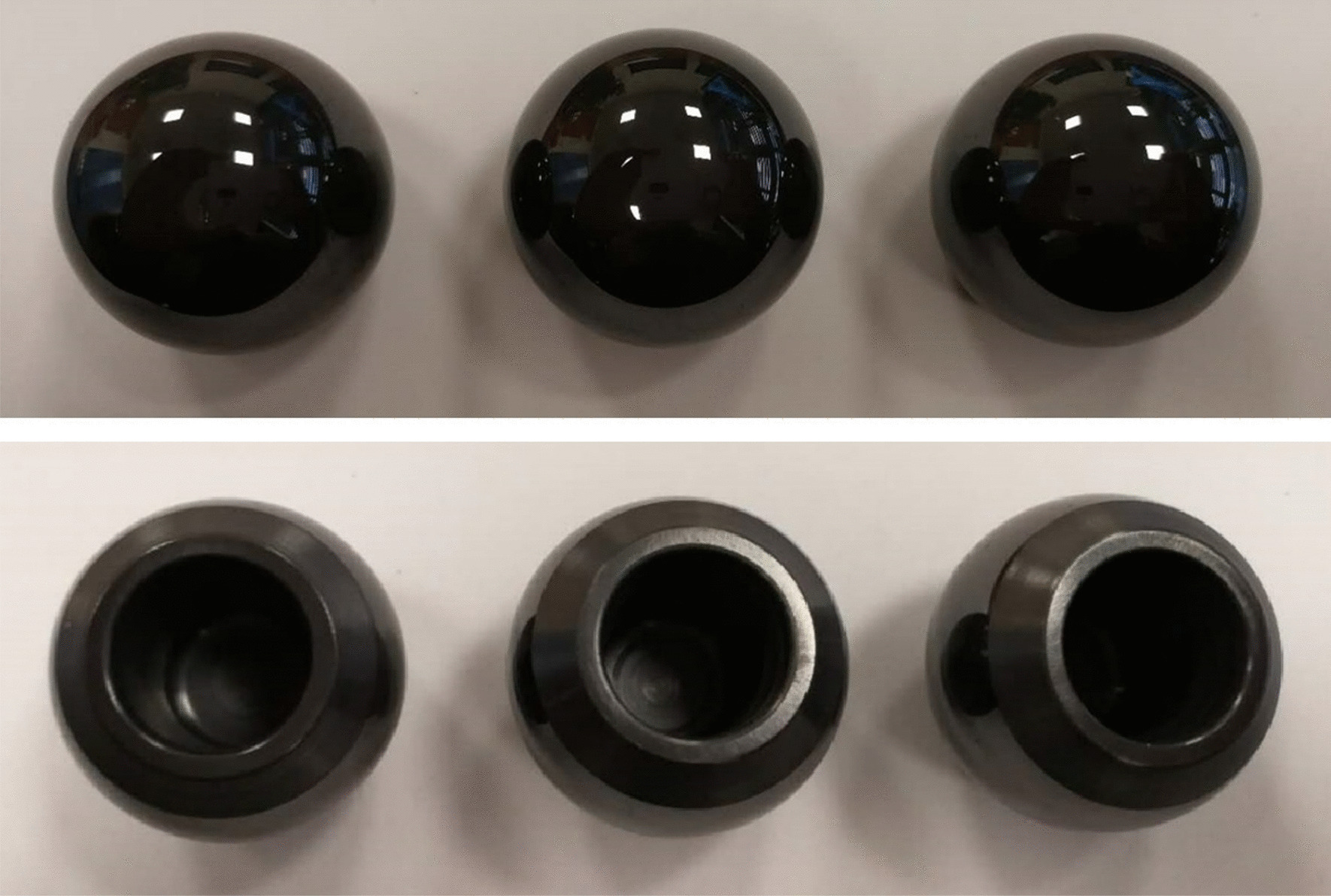
Femoral heads (Φ28 mm)

**Fig. 3:**
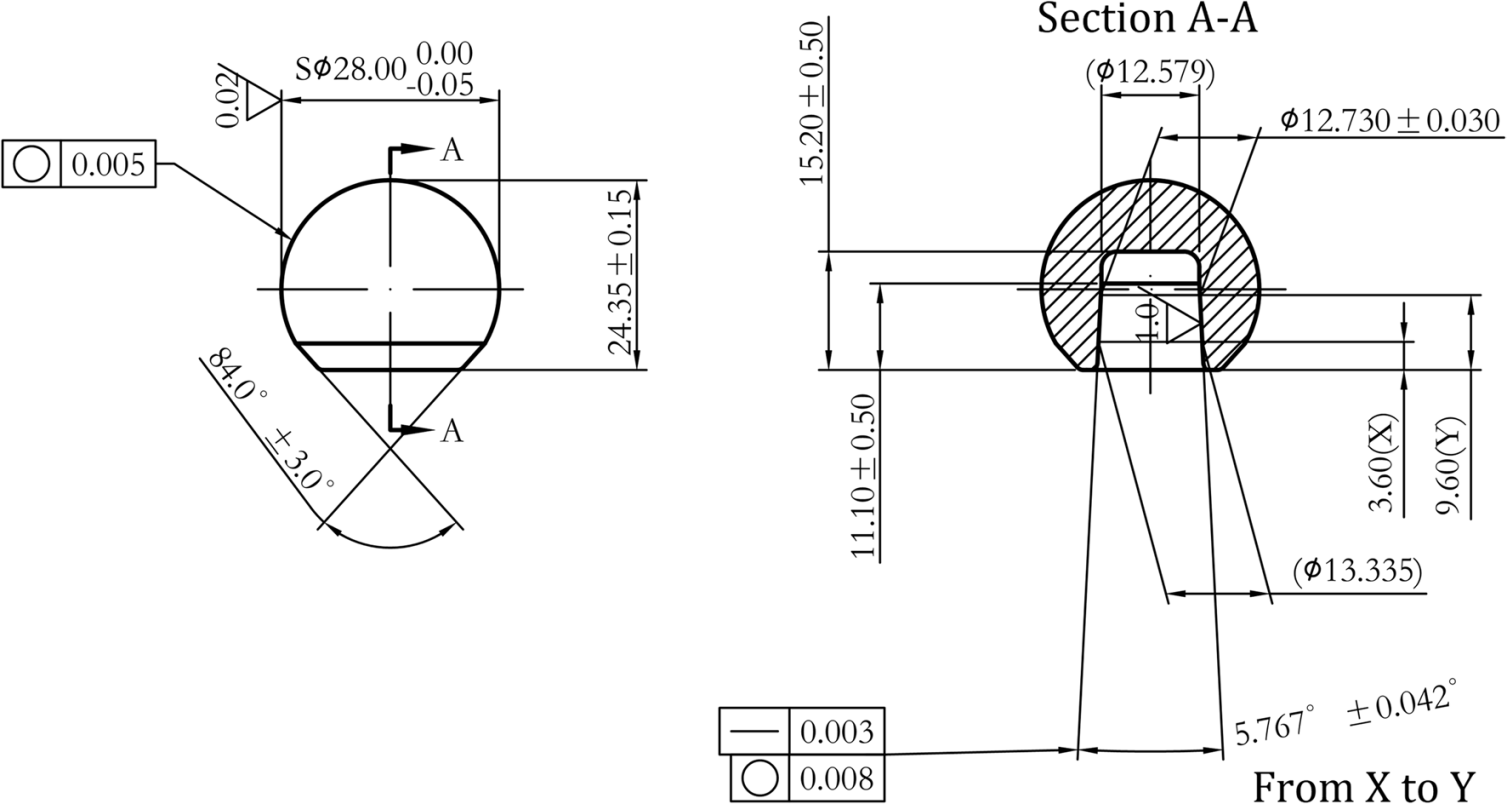
Dimension and tolerance of femoral head (Φ28 mm—Type L)

According to the specific requirements defined by the related ISO standards, the relevant silicon nitride specimens were prepared for two categories experimentation: in vitro & in vivo experiments. Both sample bars (Fig. 1) and femoral heads (Fig. 2) were produced via the patented material formula and technological process by Black Ceramic Medical Technology Co., Ltd. (Suzhou, China). The geometrical dimension and tolerance of the femoral head are detailed in Fig. 3.

**Fig. 4:**
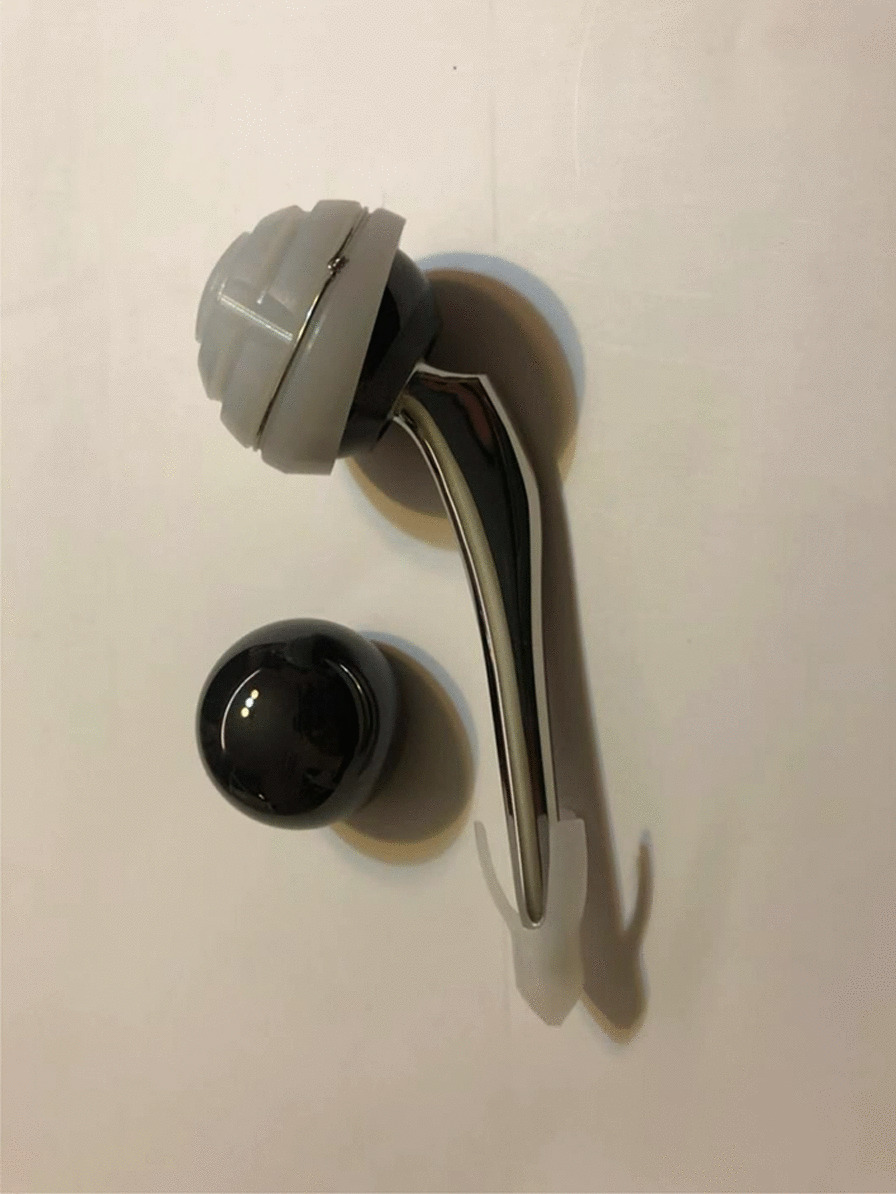
Customized total hip system for pig

As per the animal CT (Computed Tomography) scan data, the femoral stem was customized in wrought cobalt–chromium–molybdenum alloy by 3D printing from LDK Technology Co., Ltd. (Beijing, China), the cemented acetabular cup was made of UHMWPE (Fig. 4).

**Fig. 5:**
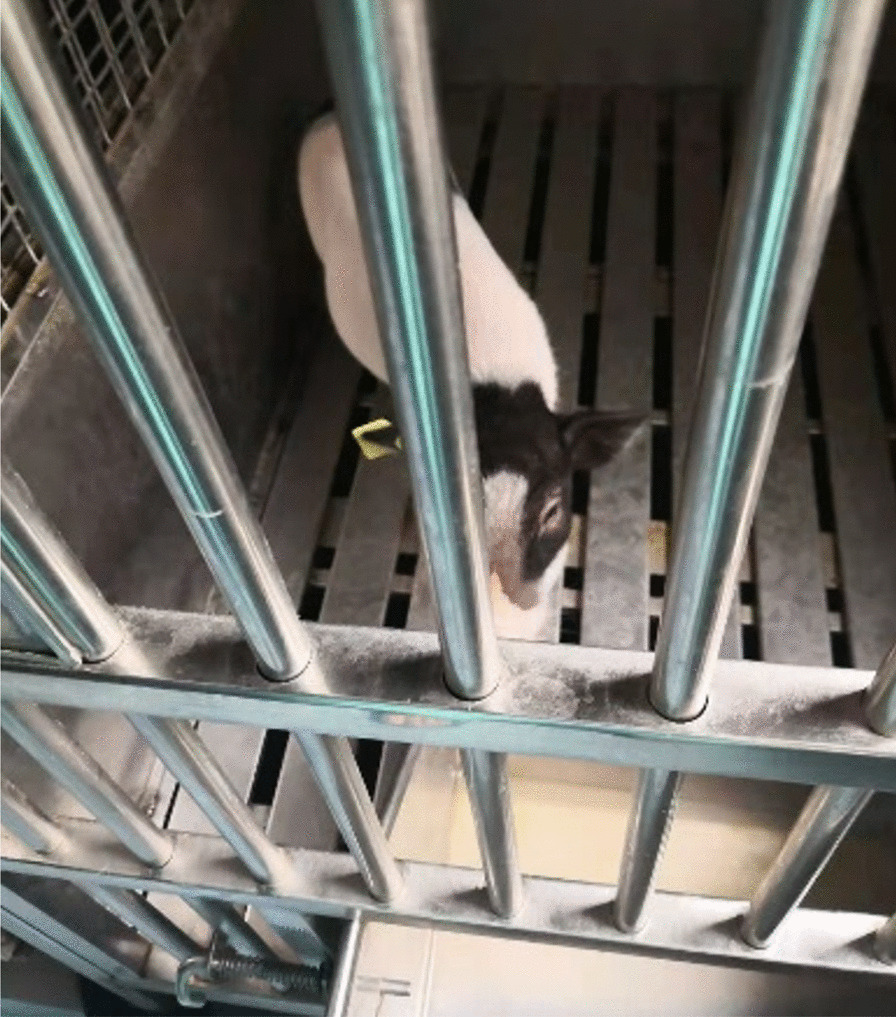
Bama minipig involved in the experiment

Thirteen health Bama minipigs (Fig. 5) were purchased from Beijing Strong Century Minipigs Breeding Base. Minipigs were fed in Laboratory Animal Center of Chinese PLA (People's Liberation Army of China) General Hospital (Beijing, China), with a 12:12-h light–dark cycle, room temperature maintained between 20 and 22 °C and humidity range 50–60%, for one week prior to experiment in order to adapt to environment. The Bama minipigs’ weight was between 25 and 29 kg. All animal care and procedures were approved by the Animal Care Committee of Chinese PLA General Hospital and complied with the latest guideline.

### In vitro experiments

#### Accelerated ageing test

Within the domain of hip implants, the accelerated ageing is usually a test method for investigating the oxidative stability of Ultra-High-Molecular-Weight-Polyethylene (UHMWPE) materials as a function of processing and sterilization method. The UHMWPE is aged at elevated temperature and at elevated oxygen pressure, to accelerate oxidation of the material and thereby allow for the evaluation of its potential long-term chemical and mechanical stability. The related principle, process and requirements are calibrated in (ISO 5834-3).

However, with regard to the ceramics applied as orthopedic prostheses, especially for the metastable oxide ceramics like zirconia or its composites, unfortunately for ageing or accelerated ageing, there is no any standard or reference on which could be relied.

Several authors reported that the ageing zirconia could be achieved by means of autoclave treatment at 134 °C and 2 bars of pressure. Under this configuration, it was calculated that 1 h exposure had theoretically the similar effect as roughly 3–4 years in the human body.

In this study, the silicon nitride sample bars (30 pieces) and eight femoral heads had been aged via 10 h autoclave treatment (134 °C, 2 bars) for the following tests.

#### Elastic moduli test

According to (ISO 17561), ten specimens unaged had been tested at room temperature for determining the Young’s modulus by sonic resonance.

#### Flexural strength test

As per (ISO 14704), ten specimens unaged with another ten specimens aged by using process defined in Sect. 2.2.1 had been investigated correspondingly for obtaining the average flexural strength in four-point-1/4 point flexure with 40 mm span semi-articulating fixtures and the bearings were free to roll.

#### Fracture toughness test

Considering the material proprieties, the notch feasibility and accuracy, the indentation fracture method had been adopted for evaluating the fracture resistance of silicon nitride. In accordance with (ISO 21618), ten specimens unaged and aged had been examined, respectively, with an indentation load of 98.07 N.

#### Fatigue strength test

As a pass/fail test for determination of the overall fatigue behavior, consistent with (ISO 22214), five specimens unaged and aged had been carried out separately at room temperature in air (25 ± 5 °C, RH40 ± 10%). The four-point-1/4 point flexure with 40 mm span semi-articulating fixtures had been used. The waveform of loading stress is a sinusoidal wave of 10 Hz frequency and a stress ration R = 0.1. The minimum and maximum force applied were 50 N and 500 N, respectively. The number of cycles for suspension was set at 10 million.

#### Vickers hardness test

Refer to (ISO 14705), five specimens unaged and aged had been inspected respectively for determining the Vickers hardness at room temperature in air. The test force used was 98.07 N.

#### Cytotoxicity test

According to (ISO 10993-5), in this study, the end-point measured in cytotoxicity determination was the measurement of cell growth via the extract test. The related materials and methods were detailed in the “Appendix” due to the constraint on length of body text.

#### Salmonella typhimurium reverse mutation assay/Ames test (OECD—Organization for Economic Co-operation and Development 471)

As per (ISO 10993-3) and OECD 471 guideline, the specimen’s genotoxicity, gene mutations in bacteria, its mutagenicity had been investigated via the number of colonies observed. The related materials and methods were detailed in the “Appendix” due to the constraint on length of body text.

#### TK gene mutation test using mouse lymphoma cells (OECD 476)

Refer to (ISO 10993-3) and OECD 476 guideline, the specimen’s genotoxicity, gene mutations in mammalian cells, had been evaluated via the observation of mouse lymphoma cells (L5178Y TK^±^-3.7.2C)’ forward mutation. The related materials and methods were detailed in the Appendix due to the constraint on length of body text.

#### Mammalian chromosome aberration test (OECD 473)

In accordance with (ISO 10993-3) and OECD 473 guideline, the specimen’s genotoxicity, clastogenicity in mammalian cells, had been examined via the inducement of chromosome aberration in the Chinese Hamster Lung (CHL) cells. The related materials and methods were detailed in the Appendix due to the constraint on length of body text.

### In vivo experiments

#### Acute and sub chronic systemic toxicity test

According to (ISO 10993-11), for the acute systemic toxicity test, 20 mice randomized in 4 groups had been involved, the related materials and methods were detailed in the “Appendix” due to the constraint on length of body text.

#### Irritation test

As per (ISO 10993-10), with regard to the test material, its potential to produce irritation had been assessed via the animal skin irritation test. The related materials and methods were detailed in the “Appendix” due to the constraint on length of body text.

#### Delayed-type hypersensitivity test

Refer to (ISO 10993-10), for the silicon nitride in this study, its delayed-type hypersensitivity had been evaluated via the guinea pig maximization test. The related materials and methods were detailed in the Appendix due to the constraint on length of body text.

#### Test for local effects after implantation in bone

In accordance with (ISO 10993-6), the local effects after implantation had been investigated via the implantation test in rabbit femur with cylindrical implants 2 mm in diameter and 6 mm in length. The related materials and methods were detailed in the “Appendix” due to the constraint on length of body text.

#### Total hip implantation test in pigs

Three mini pigs were randomly chosen and involved in preliminary experiment for being proficient in approach and surgical technique. And then, ten Bama minipigs were randomly divided into the test group (with total hip arthroplasty) and control group (with arthrotomy) regardless the gender.

**Fig. 6:**
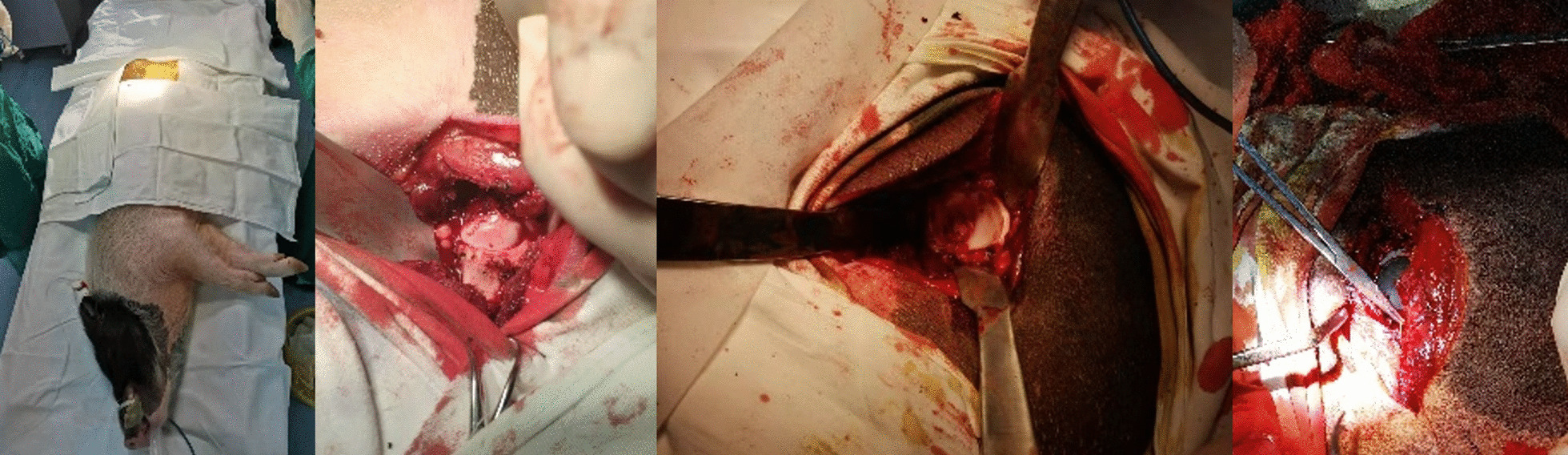
Total hip implantation in pig. From left to right: lateral position, disinfection & draping—hip joint exposure—acetabular preparation—prosthesis implantation

Before surgery, a CT scan was accomplished for ensuring its anatomical status. The general anesthesia and trachea cannula were employed. The operating side was randomized on hind legs, a lateral approach was adopted. After disinfection and draping, lateral muscles were cut to expose the hip joint. Then, a swing saw was used to cut off femoral neck, then the femoral head was removed and the acetabulum was exposed. The appropriate acetabular reamer was used to clear acetabulum. And a dislocation hook was used to lift femur and medullary space was prepared. After these preparation, acetabular cup impacted and femoral stem inserted, they were fixed by bone cement. The silicon nitride ceramic femoral head was implanted subsequently. After testing the stability of the joint, the incision was closed layer by layer. The blank control group finished arthrotomy with the same approach (Fig. 6).

After surgery, in order to reduce the risk of blood loss, infection, and pain release, tranexamic acid (1 g 100 ml^−1^ *per os*), ceftriaxone sodium (0.25 g intramuscular injection), and pethidine (2 mg kg^−1^ intramuscular injection) were applied. The clinical observations had been made weekly, the hematology and clinical biochemical determination on blood had been analyzed and recorded monthly. The second CT scan had been accomplished just before the animal euthanasia which was carried out at the end of three months after the implantation, whereafter, the pathology examination had been conducted.

## Results

According to the related ISO standards, by using the materials and methods detailed in Sect. 2, the results obtained are summarized in the relevant tables and paragraphs hereunder.

### In vitro experiments

#### Elastic moduli test

**Table:** 

Specimen no	Young’s modulus (GPa)
01	296
02	301
03	300
04	302
05	296
06	294
07	302
08	302
09	301
10	297
Mean	299
SD	3

As a comparable reference, the current mainstream product, BIOLOX® delta, from CeramTec, its Young’s modulus should be ≥ 320 GPa according to ISO 6474-2 for Type X: extra-high strength. And, the lower elastic modulus is usually considered as a favorable factor for stress shielding reduction.

#### Flexural strength test

**Table:** 

Specimen no. (unaged)	Flexural strength (MPa)	Specimen no. (aged)	Flexural strength (MPa)
11	711	21	862
12	790	22	782
13	853	23	741
14	815	24	733
15	813	25	825
16	725	26	808
17	854	27	724
18	756	28	790
19	875	29	776
20	770	30	840
Mean	796	Mean	788
SD	53	SD	44

As per ISO 6474-2, for BIOLOX® delta, the mean 4-point flexural strength should be ≥ 750 MPa and 1000 MPa for Type S: standard high strength and Type X: extra-high strength respectively. It is worthwhile to mention that the sample bar’s geometry, tolerance and surface condition including the chamfer and rounding are all crucial to the testing results. Around 1000 MPa had been registered in another research project.

#### Fracture toughness test

**Table:** 

Specimen no. (unaged)	Fracture toughness (MPa m^½^)	Specimen no. (aged)	Fracture toughness (MPa m^½^)
31	6.05	41	6.35
32	6.40	42	6.21
33	6.33	43	6.29
34	6.16	44	6.27
35	6.19	45	6.36
36	6.29	46	6.12
37	6.25	47	6.16
38	6.18	48	6.24
39	6.36	49	6.26
40	6.26	50	6.30
Mean	6.25	Mean	6.26
SD	0.10	SD	0.07

According to ISO 6474-2, for BIOLOX® delta, the fracture toughness should be ≥ 3.5 and 4.0 MPa m^½^ for Type S and Type X respectively.

#### Fatigue strength test

On conditions detailed in Sect. 2.2.5, both unaged and aged specimens passed the 10 million loading cycles without any failure or intergroup difference either.

#### Vickers hardness test

**Table:** 

Specimen no. (unaged)	Hardness (HV10)	Specimen no. (aged)	Hardness (HV10)
61	1426	66	1426
62	1460	67	1436
63	1460	68	1460
64	1449	69	1426
65	1460	70	1467
Mean	1451	Mean	1443
SD	13	SD	17

In the light of ISO 6474-2, for BIOLOX® delta, the hardness, Vickers HV1, should be ≥ 15.5 and 16.0 GPa for Type S and Type X, respectively.

#### Cytotoxicity test

On conditions detailed in Sect. 2.2.7, based upon the comparison with the positive, negative and reagent controls, the relative cell proliferation had been investigated and the qualitative evaluation had been carried out as well. Microscopically, in the majority of cells, there was no changes in general morphology, vacuolization, detachment and membrane integrity. Only few cell lysis had been observed. The cytotoxicity scale was consequently scored as 1, mildly cytotoxic, which is totally acceptable for the intended use.

#### Salmonella typhimurium reverse mutation assay/Ames test (OECD 471)

On conditions detailed in Sect. 2.2.8, comparing with the reagent controls, the inhibition of 5 types of salmonella typhimurium had not been observed in the test groups with polar or non-polar solvents. And therefore the specimen’s genotoxicity, gene mutations in bacteria, was negative.

#### TK gene mutation test using mouse lymphoma cells (OECD 476)

On conditions detailed in Sect. 2.2.9, the reagent controls was used as the baseline, with regard to the mouse lymphoma cells forward mutation, the mutant frequency ratio of the test groups with polar or non-polar solvents and the reagent controls was inferior to 2. And, the specimen’s genotoxicity, gene mutations in mammalian cells, was consequently negative.

#### Mammalian chromosome aberration test (OECD 473)

On conditions detailed in Sect. 2.2.10, according to the response of reagent controls, regarding the chromosome aberration in CHL cells, there was no statistical difference (*p* > 0.05) between the test groups and the reagent controls. And accordingly, the specimen’s genotoxicity, clastogenicity in mammalian cells, was negative.

### In vivo experiments

#### Acute & sub chronic systemic toxicity test

Under the circumstances detailed in Sect. 2.3.1, regardless of the test groups with polar/non-polar solvents or reagent controls, the clinical observation was all normal for each mouse at the different inspection points. And during the observation period, all the body weights were increased and the mortality was zero. In one word, the specimen’s acute systemic toxicity was negative.

For the subchronic systemic toxicity, in accordance with the reagent controls, in terms of the clinical observation, bodyweight change, hematology, clinical biochemical determination on blood, gross pathology and histopathology, there was no statistical difference (*p* > 0.05) between the test and control groups. As a result of which, the specimen’s subchronic systemic toxicity was negative.

#### Irritation test

Under the circumstances detailed in Sect. 2.3.2, irrespective as to whether test groups with polar/non-polar solvents or reagent controls, no any erythema or eschar or edema occurred, upon which, the specimen’s response to irritation test was negative.

#### Delayed-type hypersensitivity test

Under the circumstances detailed in Sect. 2.3.3, regardless of the test groups with polar/non-polar solvents or reagent controls, the clinical observation was all normal for each Hartley albino guinea pig during the observation period. There was no any erythema or edema observed. Based upon the patch test reactions, according to the Magnusson & Kligman scale, the grading score obtained was less than 1. Consequently, the specimen’s response to delayed-type hypersensitivity test was negative.

#### Test for local effects after implantation in bone

Under the circumstances detailed in Sect. 2.3.4, for both the test and control group, there was no abnormal local effects observed through the 26 weeks after the implantation. 1 week after implantation, a few fibrous osteoid callus were similarly observed around the test and control sample. 4 weeks after the implantation, the test and control specimen engaged closely with bone tissue, cell proliferation and trabeculae were observed equally. 26 weeks after the implantation, the new bone formed healthily around the test and control sample, the densified cortical bone were observed, there was no any foreign body reaction. In conclusion, the specimen’s response to local effects after implantation in bone was negative.

#### Total hip implantation test in pigs

**Fig. 7:**
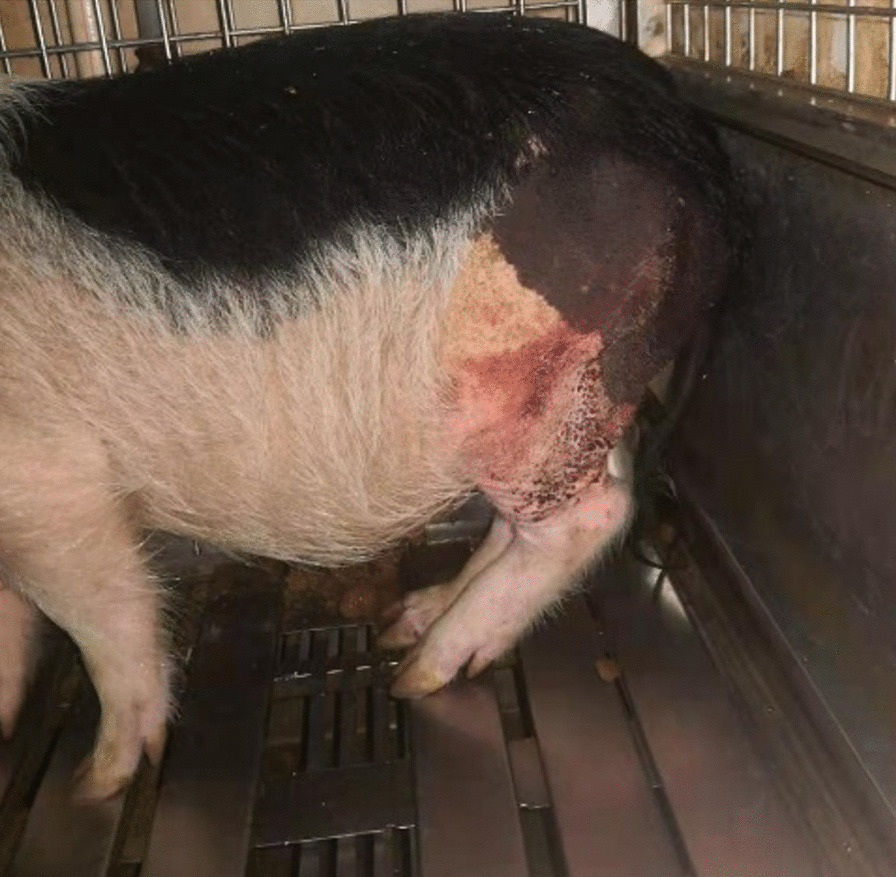
Postoperative posture

**Fig. 8:**
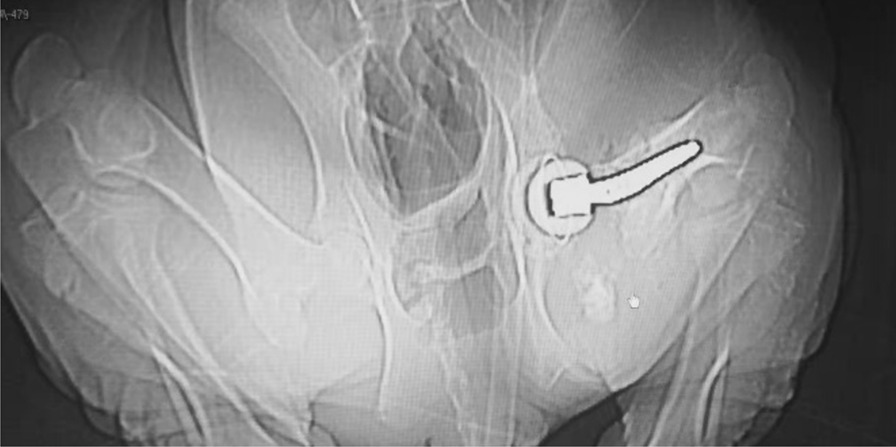
CT scan result (test group, No. 4 pig), prone position

Under the circumstances detailed in Sect. 2.3.5, comparing with the control group, all the samples in test group were functioning well through the observation period (Fig. 7). The blood test results were normal, in terms of the C-reactive protein and erythrocyte sedimentation rate, there was no difference between the test and control group. The CT scan results reveal the prostheses were on good position (Fig. 8). And the pathology examination results were negative. More detailed information could be found in another clinical report.

## Discussion

This study presents a thorough scenograph, from material level to product level, of the specific silicon nitride used as the femoral head in total hip implant system.

Comparing with the current monopolized product, BIOLOX® delta femoral head from CeramTec (Plochingen, Germany), this ZTA product’s performance is regularized by (ISO 6474-2), Black Ceramic® silicon nitride femoral head has the comparable mechanical performance.

Meanwhile, the latter’s fracture toughness is even better, the density is lower, the related implant will be lighter consequently, and exclusively, without the concern about the zirconia’s aging and instability, which are caused by the inherent and inevitable phase transformation.

Based upon the current test results obtained, the silicon nitride in this study has a superior oxidation resistance since its mechanical performances are almost indistinguishable between the unaged and aged group.

In terms of the limitation of this study, although the accelerated ageing method adopted will permit an investigator to compare the oxidative stability of silicon nitride, it is recognized that it may not precisely simulate the degradative mechanisms for an implant during real time shelf ageing and implantation. And, concerning shelf ageing component, the package environment other than air is not taken into account, the related effect could be possibly not negligible.

In addition, for the ZTA or BIOLOX® delta, according to the ISO 6474-2, after autoclaving (10 h, 0.2 MPa, 134 °C), the 20% strength degradation is allowable. For the future studies, comparing with this silicon nitride, it will be interesting to know their performances evolution with higher pressure, temperature and longer ageing process. It is important to emphasize that, comparing with the metastable oxide ceramics, the silicon nitride could withstand more severe ageing method without performance degradation due to its unique, innate and outstanding integrated characteristics.

Another gap needs to be filled, in the orthopedic domain, the implant’s wear performance is crucial as well. Although the silicon nitride is well known as a self-lubricated material, its wear resistance as the hip joint prosthesis still needs to be demonstrated in vitro according to ISO 14242 which is also the regulatory prerequisite. This will be the next step in the near future.

There are several simulator studies have been accomplished [13–15]. Recently, Affatato et al*.* accomplished the first in vitro wear behavior comparison between silicon nitride and ZTA head, the results are very promising [16].

With regard to the first ceramic total hip replacement in big animal, this test is a complementary validation of the safety and effectiveness of this silicon nitride ceramic, three months observation, it is definitely not long enough for investigating its wear performance in vivo, which is the reason for why to continue the animal study and to deploy the appropriate human clinical trial as well for the future researches.

However, in the context of (ISO 14242-1), among the current simulated conditions, it is worthwhile to investigate the more realistic wear performance under the slow-speed, high-loads with shock and interrupted (stop-start) conditions, in addition, more important, the edge loading, ageing and wearing are occurred simultaneously [17]. In this respect, beyond the metastable oxide ceramics, along the echelon of advanced technical ceramics, looking at, the non-oxide, real stable ceramic, the silicon nitride, its potential and advantages will be highlighted contrastively in the future investigations.

Furthermore, G. Pezzotti et al*.* revealed that the oxidation rate of polyethylene liners was greater when coupled with oxide as opposed to non-oxide ceramic heads [18–20]. And, in addition, J. Webster et al*.* discovered for the first time that without the use of antibiotics, the bacteria colonization was reduced and bone formation was increased on the silicon nitride surface compared with titanium and PEEK [21–23]. These two lighthouses illumine also the meaningful directions for the future exploration.

## Conclusion

Through all these in vitro & in vivo experiments, this silicon nitride hip implant’s safety and effectiveness have been investigated.

Comparing with the present metastable oxide ceramics, silicon nitride has the right combination of high strength, high hardness, excellent fracture toughness, lower density, better wear resistance, good biocompatibility, inherent stability, corrosion resistance and bioactivity, bone integration capability, all of which are desirable in medical implants.

Due to the excellent combination of properties, this silicon nitride will be an excellent material that can withstand the toughest conditions in the most demanding applications like in orthopedic and beyond.

## Figures and Tables

**Fig. 9:**
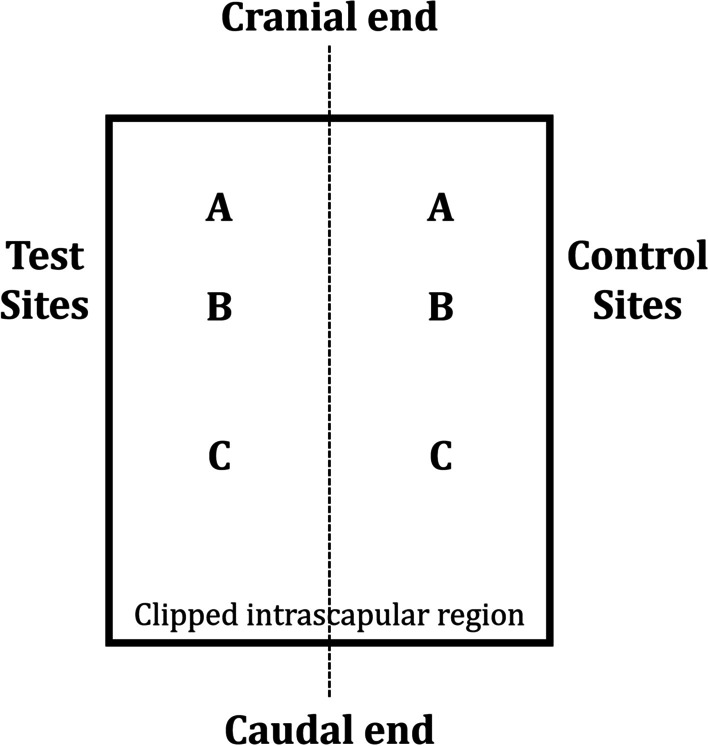
Location of intradermal injection sites

## Data Availability

The datasets generated and/or analyzed during the current study are not publicly available due to the data also forms part of an ongoing study, but are available from the corresponding author on reasonable request.

## Appendix: Test materials and methods used

### Cytotoxicity test

According to (ISO 10993-5), in this study, the end-point measured in cytotoxicity determination was the measurement of cell growth via the extract test. For the extraction vehicle, the solvent used was the culture medium with 10% calf serum. The extraction condition was 24 ± 2 h with 60 rpm agitation at 37 ± 1 °C with 2.3 g specimen in 11.5 ml culture solution. About 10% dimethyl sulfoxide (DMSO) solution was selected as the positive control. The negative control material was polyethylene, its extraction condition was 24 ± 2 h with 60 rpm agitation at 37 ± 1 °C by an interfacial area-volume ratio of 6 cm^2^ ml^−1^ culture solution. The extraction vehicles without test material subjected to the same extraction conditions were used as the reagent control. The cell lines used were American Type Culture Collection CCL 1 (NCTC-National Collection of Type Cultures clone 929). Incubation time was 72 h (5% CO_2_, 37 °C).

### Salmonella typhimurium reverse mutation assay/Ames test (OECD 471)

As per (ISO 10993-3) and OECD 471 guideline, the specimen’s genotoxicity, gene mutations in bacteria, its mutagenicity had been investigated via the number of colonies observed. The salmonella typhimurium TA97, TA98, TA100, TA102 and TA1535 had been used in this study. In terms of the extraction vehicle, the solvents used were 0.9% sodium chloride (SC) and DMSO. The extraction conditions were 1 ± 0.1 h with 60 rpm agitation at 121 ± 2 °C with 6 g specimen in 30 ml SC injection and 72 ± 2 h with 60 rpm agitation at 37 ± 1 °C with 2.8 g specimen in 14 ml DMSO solution. The extraction vehicles without test material subjected to the same extraction conditions were used as the reagent control. Incubation time was 72 h (5% CO_2_, 37 °C).

### TK gene mutation test using mouse lymphoma cells (OECD 476)

Refer to (ISO 10993-3) and OECD 476 guideline, the specimen’s genotoxicity, gene mutations in mammalian cells, had been evaluated via the observation of mouse lymphoma cells (L5178Y TK^±^-3.7.2C)’ forward mutation. With regard to the extraction vehicle, the solvents used were 0.9% SC injection and DMSO solution. The extraction conditions were 1 ± 0.1 h with 60 rpm agitation at 121 ± 2 °C with 6 g specimen in 30 ml SC injection and 72 ± 2 h with 60 rpm agitation at 37 ± 1 °C with 2.8 g specimen in 14 ml DMSO solution. The extraction vehicles without test material subjected to the same extraction conditions were used as the reagent control. The calf serum and RPMI (Roswell Park Memorial Institute)-1640 had been used as medium. Incubation time was 14 days (5% CO_2_, 37 °C).

### Mammalian chromosome aberration test (OECD 473)

In accordance with (ISO 10993-3) and OECD 473 guideline, the specimen’s genotoxicity, clastogenicity in mammalian cells, had been examined via the inducement of chromosome aberration in the Chinese hamster lung (CHL) cells. Regarding the extraction vehicle, the solvents used were 0.9% SC injection and DMSO solution. The extraction conditions were 1 ± 0.1 h with 60 rpm agitation at 121 ± 2 °C with 6 g specimen in 30 ml SC injection and 72 ± 2 h with 60 rpm agitation at 37 ± 1 °C with 2.8 g specimen in 14 ml DMSO solution. The extraction vehicles without test material subjected to the same extraction conditions were used as the reagent control. The calf serum and RPMI-1640 had been used as medium. Incubation time was 78 h (5% CO_2_, 37 °C) totally.

### Acute and sub chronic systemic toxicity test

According to (ISO 10993-11), for the acute systemic toxicity test, 20 mice randomized in 4 groups had been involved, initial weight between 17 and 23 g, aged between 6 and 8 weeks. For the extraction vehicle, the solvents used were 0.9% SC injection and cottonseed oil (CSO). The extraction conditions were 1 ± 0.1 h with 60 rpm agitation at 121 ± 2 °C with 2.4 g specimen in 12 ml SC and 2.3 g specimen in 11.5 ml CSO. The extraction vehicles without test material subjected to the same extraction conditions were used as the reagent control. The routes of administration were intravenous (IV) for SC groups and intraperitoneal (IP) for CSO groups, respectively. The dosage level was 50 ml kg^−1^. The clinical observations had been made immediately after dosing and 4 h, 24 h, 48 h and 72 h. The body weight measurements had been accomplished at 24 h, 48 h and 72 h correspondingly.

With regard to the sub chronic systemic toxicity test, as per the sex, 40 rats randomized in 4 groups had been involved, initial weight range was 191–215 g for female and 211–239 g for male, age range 6–9 weeks. For the extraction vehicle, the solvent used was 0.9% SC injection. The extraction condition was 72 ± 2 h with 60 rpm agitation at 37 ± 1 °C with a ratio of 0.2 g ml^−1^. The extraction vehicles without test material subjected to the same extraction conditions were used as the reagent control. The route of administration was IV in a daily basis for consecutive 28 days. The dosage volume was 10 ml kg^−1 ^* BW-Body Weight. The clinical observations had been made every day, the body weight had been recorded weekly. The hematology and clinical biochemical determination on blood, gross pathology and histopathology had been carried out at the end of the test period.

### Irritation test

As per (ISO 10993-10), with regard to the test material, its potential to produce irritation had been assessed via the animal skin irritation test. Three healthy young adult albino rabbits had been randomly chosen and used. For the extraction vehicle, the solvents used were 0.9% SC injection and CSO. The extraction conditions were 1 ± 0.1 h with 60 rpm agitation at 121 ± 2 °C with 2.8 g specimen in 14 ml SC and 3.2 g specimen in 16 ml CSO. The extraction vehicles without test material subjected to the same extraction conditions were used as the reagent control. At the cranial end, five sites, on each side of the spine, had been selected for SC group and its control. And for the same animal, at the caudal end, 10 sites in total had been selected for CSO group and its control on both sides of the spine. The dosage volume was 0.2 ml at each test and control site by intradermal injection. The clinical observations had been made immediately after dosing and 24 ± 2 h, 48 ± 2 h and 72 ± 2 h. The potential erythema or eschar or edema should be recorded correspondingly.

### Delayed-type hypersensitivity test

Refer to (ISO 10993-10), for the silicon nitride in this study, its delayed-type hypersensitivity had been evaluated via the guinea pig maximization test. 30 Hartley albino guinea pigs, bodyweight range from 300 to 400 g, had been randomly chosen and involved. For the extraction vehicle, the solvents used were 0.9% SC injection and CSO. The extraction conditions were 1 ± 0.1 h with 60 rpm agitation at 121 ± 2 °C with 2 g specimen in 10 ml SC or CSO. The 1-Chloro-2, 4-dinitrobenzene was selected as the positive control, and the extraction vehicles without test material subjected to the same extraction conditions were used as the reagent control.

For the intradermal induction phase, a pair of 0.1 ml of each of the following, into each animal, had been made at the injection sites (A, B and C) as shown in Fig. 9 in the clipped intrascapular region.

Site A: a 50:50 (volume ration) stable emulsion of Freund’s complete adjuvant (FCA) mixed with the chosen solvent.

Site B: the test sample (undiluted extract), the solvent alone for the control animals.

Site C: the test sample at the concentration used at site B, emulsified in a 50:50 (volume ration) stable FCA emulsion and the chosen solvent (50%), the emulsion of the blank liquid with FCA for the control animals.

Seven days after completion of the intradermal induction phase, the topical induction phase was started by using a patch of area approximately 8 cm^2^ filter paper (soaked in the concentration selected for site B) to cover the intradermal injection sites. These area had been pretreated with 10% sodium dodecyl sulfate massaged into the skin 24 ± 2 h before the patch was applied. The patches were secured with an occlusive dressing and these dressing and patches were removed after 48 ± 2 h. The control animals were treated similarly by using the reagent control alone.

At 14 days after completion of the topical induction phase, the challenge phase was kicked off by using the patches (soaked in the concentration selected for site C) on the upper flank of each animal. After 24 ± 2 h, these patches and dressings were removed.

24 h and 48 h after the removal of the dressings, the appearance of the challenged skin sites of the test and control animals were observed and the skin reactions for erythema and edema were recorded according to the Magnusson & Kligman grading.

### Test for local effects after implantation in bone

In accordance with (ISO 10993-6), the local effects after implantation had been investigated via the implantation test in rabbit femur with cylindrical implants 2 mm in diameter and 6 mm in length. The UHMWPE bars with same shape were used as the control implants. There were twenty rabbits randomly chosen and involved in this study. Two implantation sites were chosen on each rabbit femur and the control specimens were implanted contralaterally in the equivalent anatomical sites. The animal euthanasia was carried out respectively at 1 week, 4 weeks and 26 weeks after the implantation, and retrieval of the implant together with sufficient unaffected surrounding tissue. The relevant biological response was evaluated by documenting the macroscopic and histopathological responses as a function of time.

## Abbreviations

ISO: International Organization for Standardization
THA: Total Hip Arthroplasty
FDA: U. S. Food and Drug Administration
ZTA: Zirconia Toughened Alumina
3Y-TZP: Yttria-stabilized Tetragonal Zirconia Polycrystal
CT: Computed Tomography
PLA: People's Liberation Army of China
UHMWPE: Ultra-High-Molecular-Weight-Polyethylene
OECD: Organization for Economic Co-operation and Development
CHL: Chinese Hamster Lung
DMSO: Dimethyl Sulfoxide
NCTC: National Collection of Type Cultures
SC: Sodium Chloride
RPMI: Roswell Park Memorial Institute
CSO: Cottonseed Oil
IV: Intravenous
IP: Intraperitoneal
BW: Body Weight
FCA: Freund's Complete Adjuvant
